# Stool Xpert^®^ MTB/RIF test for the diagnosis of childhood pulmonary tuberculosis at primary clinics in Zimbabwe

**DOI:** 10.5588/ijtld.16.0357

**Published:** 2016-12-15

**Authors:** M. Chipinduro, K. Mateveke, B. Makamure, R. A. Ferrand, E. Gomo

**Affiliations:** * University of Zimbabwe College of Health Sciences, Medical Laboratory Sciences, Harare, Zimbabwe; † University of Zimbabwe College of Health Sciences, Research Support Centre, Harare, Zimbabwe; ‡ Biomedical Research and Training Institute, Harare, Zimbabwe; § Clinical Research Department, London School of Hygiene & Tropical Medicine, London, UK; ¶ Traditional Medical Laboratory, University of KwaZulu-Natal, Durban, South Africa

**Keywords:** GeneXpert, faecal specimen, children

## Abstract

**OBJECTIVE:** To evaluate the diagnostic performance of Xpert^®^ MTB/RIF on stool samples from children with clinical suspicion of pulmonary tuberculosis (PTB) at primary care clinics.

**DESIGN:** A cross-sectional diagnostic evaluation enrolling 5–16 year olds from whom one induced sputum (IS) sample was tested for microbiological TB confirmation. Results of a single stool sample tested using Xpert were compared against microbiologically confirmed TB, defined as a positive result on sputum microscopy and/or culture and/or IS Xpert.

**RESULTS:** Of 222 children enrolled, 218 had complete microbiological results. The median age was 10.6 years (interquartile range 8–13). TB was microbiologically confirmed in 19/218 (8.7%) children. Of these, respectively 5 (26%), 9 (47%) and 15 (79%) were smear-, culture- and IS Xpert-positive. Stool Xpert was positive in 13/19 (68%) microbiologically confirmed cases and 4/199 (2%) microbiologically negative cases. Stool Xpert detected 76.9% (10/13) of human immunodeficiency virus (HIV) infected and 50% (3/6) of non-HIV-infected children with microbiologically confirmed TB (*P* = 0.241).

**CONCLUSION:** Stool Xpert is a potential alternative screening test for children with suspected TB if sputum is unavailable. Strategies to optimise the diagnostic yield of stool Xpert assay need further study.

## RESUME

**OBJECTIF :** Evaluer la performance diagnostique du test Xpert^®^ MTB/RIF sur des échantillons de selles d'enfants avec suspicion clinique d'avoir une tuberculose (TB) pulmonaire dans des structures de soins de santé primaires.

**SCHÉMA :** Evaluation transversale du diagnostic enrôlant des enfants âgés de 5 à 16 ans pour lesquels un échantillon d'expectoration induite a également été testé pour une confirmation microbiologique de la TB. Les résultats d'un seul échantillon de selles testé par Xpert ont été comparés à ceux d'une TB confirmée par microbiologie, définie comme un résultat positif de la microscopie de crachats et/ou de la culture et/ou de l'Xpert sur expectoration induite.

**RÉSULTATS :** Sur 222 enfants enrôlés, 218 ont eu des résultats microbiologiques complets. L'âge median était de 10,6 ans (intervalle interquartile 8–13). Une TB a été confirmée par microbiologie chez 19/218 (8,7%) enfants. Parmi ces derniers, 5 (26%), 9 (47%) et 15 (79%), respectivement, ont eu un résultat positif de frottis, de culture et d'Xpert sur expectoration induite. L'Xpert sur échantillon de selles a été positif dans 13/19 (68%) cas microbiologiquement confirmés et 4/199 (2%) cas microbiologiquement négatifs. L'Xpert sur selles a détecté 76,9% (10/13) des enfants positifs au virus de l'immunodéficience humaine (VIH) et 50% (3/6) des enfants VIH négatifs avec une TB microbiologique (*P* = 0,241).

**CONCLUSION :** L'Xpert pourraitêtre un test de dépistage alternatif potentiel pour les enfants avec suspicion de TB si l'on ne dispose pas de crachats. Les stratégies visant à optimiser le rendement diagnostique du test Xpert sur des selles doivent bénéficier d'autres études.

## RESUMEN

**OBJETIVO:** Evaluar el rendimiento diagnóstico de la prueba Xpert^®^ MTB/RIF en las muestras de materias fecales de niños con presunción clínica de tuberculosis (TB) pulmonar, en los consultorios de atención primaria.

**MÉTODO:** Fue este un estudio transversal de evaluación del diagnóstico de TB en niños de edad de 5–15 años, de quienes se obtuvo una muestra de esputo inducido con fines de confirmación microbiológica. Se compararon los resultados de la prueba Xpert en una muestra única de materia fecal con los casos de TB confirmada microbiológicamente mediante un resultado positivo de la baciloscopia del esputo, el cultivo o la prueba Xpert en el esputo inducido.

**RESULTADOS:** De los 222 niños que participaron en el estudio, 218 contaban con resultados microbiológicos completos. La mediana de la edad fue 10,6 años (intervalo intercuartil 8–13). Se obtuvo confirmación microbiológica del diagnóstico de TB en 19/218 niños (8,7%). De estos niños, 5 obtuvieron un resultado positivo de la baciloscopia (26%), 9 un cultivo positivo (47%) y en 15 casos se observó un resultado positivo con la prueba Xpert en la muestra de esputo inducido (79%). La prueba Xpert en la muestra fecal fue positiva en 13/19 casos confirmados bacteriológicamente (68%) y en 4/199 sin confirmación microbiológica (2%). Con la prueba Xpert en muestras de materia fecal se detectó el 76,9% de los casos coinfectados por el virus de la inmunodeficiencia humana (VIH) (10/13) y el 50% de los niños sin infección por el VIH (3/6) con TB confirmada microbiológicamente (*P* = 0,241).

**CONCLUSIÓ N:** La prueba Xpert en muestras de materia fecal puede constituir otra opción de pruebas de detección en los niños con presunción clínica de TB, cuando no se cuenta con muestras de esputo. Se precisan nuevas investigaciones sobre las estrategias que puedan mejorar el rendimiento diagnóstico en estas muestras.

CHILDHOOD TUBERCULOSIS (TB) contributes 10% of the total 9 million incident cases worldwide, with an estimated 136 000 deaths among children.^1^ The childhood TB burden is largely due to undiagnosed and late diagnosis of adult TB, which creates a reservoir for transmission to children.^2^

The principal challenge to timely diagnosis of childhood TB is the difficulty in obtaining spontaneously expectorated sputum, necessitating sputum induction, which requires skill and infrastructure. Furthermore, sputum from children is often paucibacillary, as children are less likely to form cavitary lesions in lungs to contain the bacilli.^2^,^3^ The sensitivity of the standard diagnostic tests, sputum microscopy and culture, is therefore lower. A more easily accessible sample that can be obtained with minimal skill at primary health care level and an alternative diagnostic tool is therefore required for the diagnosis of TB in children.

Mycobacterium tuberculosis DNA can be detected by an automated molecular assay, Xpert^®^ MTB/RIF (Cepheid, Sunnyvale, CA, USA). Automation includes sample processing, nucleic acid extraction, polymerase chain reaction (PCR) and the detection of specific M. tuberculosis codon sequences within a closed system.^4^,^5^ The assay is rapid, producing a result in 3 h, with little hands-on time in sample processing.

Previous studies have shown high sensitivity of Xpert when sputum,^6–8^ gastric lavage,^9^,^10^ bronchoalveolar lavage^11^ and nasopharyngeal aspirates^12^ were used. Xpert was therefore endorsed by the World Health Organization as an initial test for diagnosing TB in children. However, invasive procedures are required to obtain such samples.

Stool is a potential sample that can be collected non-invasively. Children tend to swallow sputum when they cough and M. tuberculosis DNA has been shown to survive the harsh acidic and digestive environment of the gastro-intestinal tract.^13^ We therefore evaluated the diagnostic performance of Xpert using stool in children with clinically suspected pulmonary TB (PTB) presenting to primary care clinics in Harare, Zimbabwe. The diagnostic yield of stool Xpert was assessed against confirmed microbiological PTB determined by testing induced sputum (IS) with microscopy, solid culture and Xpert.

## METHODS

### Participants

Participants were recruited from eight primary care clinics in Harare between September 2013 and October 2014, with guardian consent and participant assent. Participants were children aged 5–16 years presenting with a chronic cough of >2 weeks and any one of the classic signs and symptoms of TB, including weight loss, loss of appetite, persistent fever without an apparent cause, night sweats or history of close contact with a TB index patient, defined as living in close proximity (sharing a room within a household) with an adult diagnosed with TB within the preceding 12 months.

Those aged 0–5 years were not included. At the time of seeking ethical approval, evidence on the safety and tolerability of sputum induction in children was requested, as sputum induction is rarely practised. The available literature did not meet the satisfaction of the ethical committee to allow the procedure to be performed in children aged <5 years.

Participants were excluded if they had an already confirmed TB diagnosis, had been on anti-tuberculosis treatment for >72 h before enrolment, required emergency medical attention or were resident outside Harare.

### Study procedures

A questionnaire was used to collect data on demographics and physical examination. Human immunodeficiency virus (HIV) status was obtained from documented proof of test result. In case of unknown HIV status, participants were offered the test through the clinic's HIV testing protocols. All participants were asked to provide a stool sample and to undergo sputum induction to obtain one sputum sample.

Sputum induction was performed by a trained nurse following the method described by Zar et al.,^14^ except where it was contraindicated.^15^ The procedure was performed in an open area after a 2–3 h fast. Two doses of 100 μg salbutamol were administered via an aerochamber to prevent bronchoconstriction, followed by nebulisation with 5% hypertonic saline for 5 min, or until the participant started to cough. Oxygen saturation was monitored during and 30 min after the procedure. IS was collected in a sterile container by nasopharyngeal suction, if required. If the participant failed to produce sputum, the procedure was repeated once, 5 min after the first attempt.

The stool sample (about 5 g) was collected into a wide mouthed specimen collection jar on the spot or submitted the following day. Samples were transported at 4–8°C to the laboratory within 8 h of collection.

Anti-tuberculosis treatment was commenced by the residing medical officer at the clinic following Zimbabwe's national TB guidelines, in which diagnosis is based on clinical history and examination, chest radiography (CXR) and/or microbiological results. Study test results on microbiological confirmation were made available to the medical officer to assist in participant management.

### Laboratory methods

Sputum was processed for microscopy, culture and Xpert testing. It was decontaminated by adding an equal volume of 4% sodium hydroxide for 15 min, neutralised with phosphate buffered saline (PBS) and centrifuged at 3200× *g* for 20 min to obtain a sediment which was resuspended in 1.5 ml PBS. One drop of the sediment was used to make a smear, which was stained using the Ziehl-Neelsen (ZN) method. All smears were re-read by a second reader blinded to the first reading. Discordant results were resolved through a third reading by a different reader. IS sediment (0.1 ml) was cultured on Löwenstein-Jensen (LJ) slopes at 37°C for 8 weeks, with weekly monitoring for growth. At least one colony on LJ was considered to be positive for M. tuberculosis growth, confirmed by ZN stain and speciation using the SD Bioline TB Ag MPT64 antigen rapid test (Standard Diagnostics Inc., Kyonggi, South Korea). Discordant ZN-positive/MPT64 antigen-negative samples were speciated using colony morphology, growth at different temperatures and on para-nitrobenzoic acid-containing LJ slopes. Confirmed cultures underwent phenotypic direct drug susceptibility testing (DST) on LJ slopes for 4 weeks at 37°C. For Xpert testing, 0.5 ml of IS sediment was mixed with 1.5 ml Xpert reagent, and the mixture was tested according to the manufacturer's instructions.

Stool samples were processed within 2 days of collection, as described by Nicol et al.^16^ An aliquot of 0.15 g was measured into a centrifuge tube using a sterile disposable plastic loop that was left inside the tube; 2.4 ml PBS was then added, and the mixture was vortexed to remove stool particles from the loop. The loop was removed and the sample mixture was left undisturbed at room temperature for 20 min. Two aliquots of 1 ml supernatant were removed. One aliquot was used for immediate testing and another was stored for repeat testing if required. Before testing, the processed stool sample was centrifuged at 3200× *g* for 15 min; the sediment was then resuspended in 1 ml PBS, mixed with 2 ml Xpert reagent and tested according to the manufacturer's instructions. Interpretation of results was auto-generated by the Xpert system. Personnel carrying out the index test were blinded to sputum test results.

### Data analysis

Sample size was based on an anticipated sensitivity of the stool Xpert test of 80%, 10% level of precision and 4% anticipated loss to follow-up. During sample size computation, no data were available on TB prevalence among children in our setting; prevalence was therefore set at 29%, adopted from a community study of paediatric TB contacts in South Africa.^17^

Data were analysed using STATA, version 13.0 (StataCorp, College Station, TX, USA). Descriptive statistics were used to characterise the study population. The χ^2^ test was used to test for associations. Performance of stool Xpert was assessed as the proportion of stool Xpert-positive cases among microbiologically confirmed and clinically diagnosed TB cases. Microbiological TB was defined as a positive result on ZN smear microscopy and/or culture and/or Xpert test on IS. Indeterminate TB status results and samples without complete data on index and reference tests were excluded from the analysis. The study also aimed to evaluate the performance of stool Xpert in TB clinical case definitions as defined by Graham et al.^18^ However, the number of children who underwent CXR was too small (*n* = 24) to provide meaningful classification. According to the Zimbabwe national TB guidelines, a CXR is required for children with clinical suspicion of PTB but without microbiological confirmation; CXR data were therefore dependent on the clinic's operational systems.

Ethics approval for the study was obtained from the Harare City Health Department, Harare, the Joint Parirenyatwa Hospital and College of Health Sciences Research Ethics Committee, Harare, and the Medical Research Council of Zimbabwe, Harare, Zimbabwe.

## RESULTS

Of the total 222 participants recruited, 4 were excluded from the analysis: 1 participant was withdrawn by the guardian, 2 were unable to provide IS samples and 1 had an IS Xpert test that produced an error result despite repeated testing. The median age of the 218 remaining participants was 10.6 years (interquartile range [IQR] 8–13); 44% were male. The median *Z*-scores for weight-for-age and height-for-age were respectively −1.09 (IQR −1.99 to −0.46) and −1.88 (IQR −3.70 to −0.73). HIV status was available for 201 (91%) participants, of whom 111 (51%) were HIV-infected, with 55 (49.5%) undergoing antiretroviral therapy (ART). The distribution of demographic characteristics between microbiological TB and no TB groups is shown in Table 1.

**Table 1:**
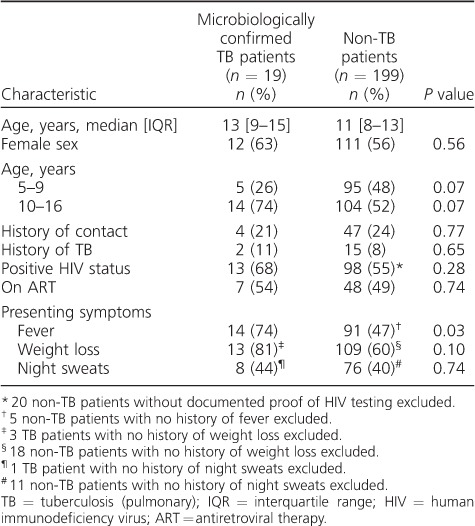
Baseline characteristics of TB and non-TB child patients (*n* = 218)

### Microbiologically confirmed TB

TB prevalence by microbiological confirmation, defined as a positive result on microscopy and/or culture and/or Xpert testing on IS, was 8.7% (19/218). Five (26%) of the 19 microbiologically confirmed TB cases were aged <10 years. Of the 19 microbiologically confirmed TB cases, 5 (26%) were smear-positive, 9 (47%) were culture-positive and 15 (79%) were Xpert-positive on IS. Among smear-positive cases, 2 (40%) were Xpert-positive, culture-positive, 1 (20%) Xpert-positive, culture-negative and 2 (40%) Xpert-negative, culture-negative. Only 2/19 (10.5%) microbiologically confirmed TB cases were positive on all three microbiological tests (Figure). Rifampicin resistance was not detected in any of the TB cases by either direct DST or Xpert testing. A positive HIV status was documented in 13/19 (68%) microbiological TB cases, 7 (54%) of whom were on ART; 11 (85%) children tested positive with IS Xpert (Figure). Thirty-two (14.7%) of 218 children were commenced on anti-tuberculosis treatment, 19 (59.4%) of whom were microbiologically confirmed and 13 (40.6%) clinically diagnosed by a physician.

**Figure:**
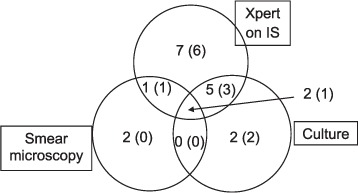
Venn diagram for positive smear microscopy, culture and Xpert on IS. This diagram includes 19 children with microbiologically confirmed tuberculosis. Numbers in brackets indicate HIV-infected cases. All the 19 microbiologically confirmed children received anti-tuberculosis treatment. IS = induced sputum; HIV = human immunodeficiency virus.

### Diagnostic performance of stool Xpert

Stool Xpert was positive in 17/218 (8.3%) children. Among HIV-infected children, stool Xpert was positive in 12.4% (14/113) of cases vs. 3.5% (3/87) in non-HIV-infected children (*P* = 0.025). Of the 17 stool Xpert-positive cases, 13 (76.5%) had microbiologically confirmed TB. Stool Xpert detected respectively 60% (3/5), 66.7% (6/9) and 86.7% (13/15) of smear-positive, culture-positive and IS Xpert-positive TB cases. Stool Xpert detected 13/19 (68%) microbiologically confirmed TB cases and was negative in 195/199 (98%) children with negative results on microbiologically tests (Table 2). Of the 19 microbiologically confirmed TB cases, stool Xpert identified 76.9% (10/13) of HIV-infected vs. 50% (3/6) of non-HIV-infected cases (*P* = 0.241), and 71.4% (10/14) of children aged ⩾10 years vs. 60% (3/5) of those aged <10 years (*P* = 0.637).

**Table 2:**
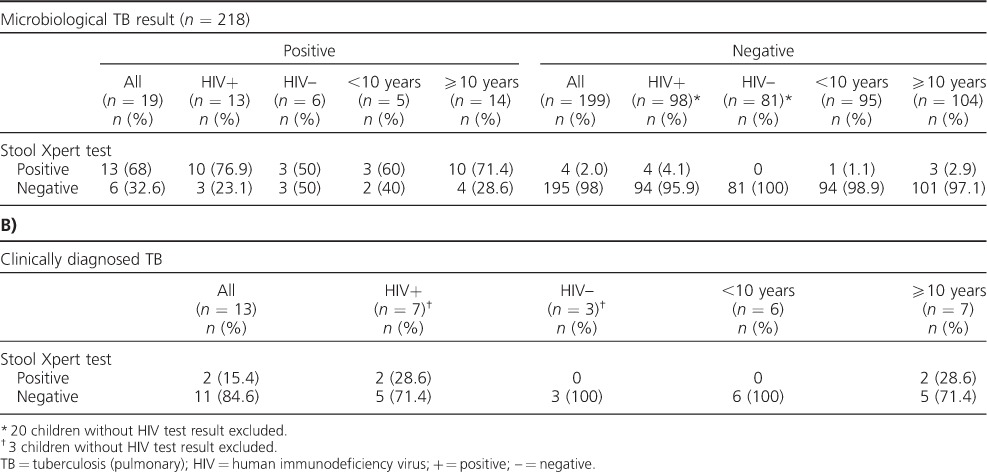
Diagnostic performance of stool Xpert in **A)** microbiologically determined TB cases and in **B)** clinically diagnosed TB cases

Four (2.0%) of the 199 children with microbiologically negative results were stool Xpert-positive (Table 2). The four children with a stool Xpert but negative IS Xpert and culture were all HIV-infected, had no history of TB and no history of contact with a TB index patient. Two (50%) of the four children received a clinical diagnosis and were the only ones with a positive stool Xpert among 13 clinically diagnosed children. Of these two children, one (50%) had clinical improvement at 2 months of anti-tuberculosis treatment (determined after the study). Of 186 children not commenced on treatment, two (1.1%) were stool Xpert-positive; clinical improvement could not be reliably determined after the study.

## DISCUSSION

The main finding of the study is that Xpert testing on stool identified 68% of microbiologically confirmed TB and was negative in 98% of children with negative microbiological results. Similar to findings by Nicol et al.,^16^ this study showed that Xpert on stool was more sensitive in HIV-infected than in non-HIV-infected children. Although the test failed to identify 32% of TB cases, it may be of more value in HIV-infected children, who run a high risk of acquiring TB, as well as high risk of disease progression. Marcy et al. reported a sensitivity of 62% for stool Xpert testing among HIV-infected children.^19^ Smear microscopy, the routinely available test at primary care centres, has a sensitivity of <10–15% in children with proven TB,^20–22^ and its performance is even poorer in those with HIV infection.^23^

Xpert is currently being rolled out in many parts of southern Africa for use as an initial test for TB diagnosis. The test offers rapid diagnosis and detects more cases than smear microscopy in children.^7–9^,^21^ However, its limitation is that it requires a sputum sample, which is difficult to obtain freely expectorated from children. At primary care level, sputum induction in children is rarely attempted, and when it is performed the quality of the specimen varies depending on the personnel, resulting in variable test sensitivity. Welday et al. showed that stool Xpert testing in children detected all of the sputum smear-positive cases.^24^ In our study, stool Xpert testing helped to identify most of the IS Xpert-positive cases, suggesting that when a sputum sample is not readily available a stool sample, which is easier to collect, may be used with the Xpert test. However, the diagnostic yield of the stool Xpert test still requires optimisation.

The inability of stool Xpert to detect six of the microbiologically confirmed cases may be due to interference by PCR inhibitors present in stool samples. Emerging evidence suggests that using larger sample volumes, of 0.6 g^25^ and 2 cm^3^,^26^ and pre-treatment with a stool-processing buffer to inactivate PCR inhibitors,^25^ achieves greater diagnostic yield of the stool Xpert test.

The stool Xpert test performed poorly in the clinically diagnosed children. Xpert testing has consistently shown poor performance in clinically defined probable TB cases in children.^27^,^28^ This is more likely to indicate a limitation of the assay's detection limit than a limitation of the sample used, as clinical microbiologically negative TB cases are more likely to have paucibacillary disease. The two clinically diagnosed children identified using stool Xpert were both HIV-infected, again indicating the potential use of the test in HIV infection.

The main limitation of our study was the lower than expected TB prevalence, which limited study power. Second, we used one stool sample collected at random; a second sample has been shown to have incremental value in detecting additional cases.^6^,^7^,^22^ Our findings cannot be generalised to all children, as the performance of the stool Xpert test was assessed only in children aged 5–16 years. Different results may occur with children aged <5 years, in whom TB disease presentation is markedly different.

In conclusion, the stool Xpert test holds promise as an alternative to sputum Xpert testing, particularly in HIV-infected children. Stool collection is easier and relatively safe compared to sputum collection, and may potentially have diagnostic utility if its sensitivity is optimised. Further studies are required to determine optimal stool collection times, optimal sample volumes, the need for a second sample, concentration techniques that allow maximum yield and test combination algorithms.
